# Molecular mapping of neuronal architecture using STORM microscopy and new fluorescent probes for SMLM imaging

**DOI:** 10.1117/1.NPh.11.1.014414

**Published:** 2024-03-08

**Authors:** Victor Breton, Paul Nazac, David Boulet, Lydia Danglot

**Affiliations:** a Université Paris Cité, Institute of Psychiatry and Neuroscience of Paris, INSERM U1266, Membrane Traffic in Healthy and Diseased Brain, Paris, France; b Université Paris Cité, Institute of Psychiatry and Neuroscience of Paris, INSERM U1266, NeurImag Core Facility, Paris, France

**Keywords:** super-resolution, distribution analysis, colocalization, synapse, membrane probe, molecular mapping

## Abstract

Imaging neuronal architecture has been a recurrent challenge over the years, and the localization of synaptic proteins is a frequent challenge in neuroscience. To quantitatively detect and analyze the structure of synapses, we recently developed free SODA software to detect the association of pre and postsynaptic proteins. To fully take advantage of spatial distribution analysis in complex cells, such as neurons, we also selected some new dyes for plasma membrane labeling. Using Icy SODA plugin, we could detect and analyze synaptic association in both conventional and single molecule localization microscopy, giving access to a molecular map at the nanoscale level. To replace those molecular distributions within the neuronal three-dimensional (3D) shape, we used MemBright probes and 3D STORM analysis to decipher the entire 3D shape of various dendritic spine types at the single-molecule resolution level. We report here the example of synaptic proteins within neuronal mask, but these tools have a broader spectrum of interest since they can be used whatever the proteins or the cellular type. Altogether with SODA plugin, MemBright probes thus provide the perfect toolkit to decipher a nanometric molecular map of proteins within a 3D cellular context.

## 1 Introduction

Imaging neuronal architecture has been a recurrent challenge over the years. The use of Golgi technique by Ramón y Cajal paved the way for the first characterization of neuronal architecture using microscopy on fixed brains. Indeed, metallic impregnation with silver salts provided an opportunity to see and reconstruct the dendritic architecture of various types of neurons in the depth of the nervous tissue. Although Golgi staining is still used in widefield microscopy, its use in confocal microscopy remains limited for 3D analysis. The use of fluorescent labeling in conjunction with 3D microscopy led to the production of large amounts of published data that are on the way to being classified and accessible through various infrastructures or free repositories (eBrains, Zenodo, etc.). This huge amount of data and their accessibility raise the question of potential new automated, unbiased statistical analysis.

## 2 Colocalization and Coupling Analysis in Conventional and Super-Resolution Microscopy

**Fig. 1:**
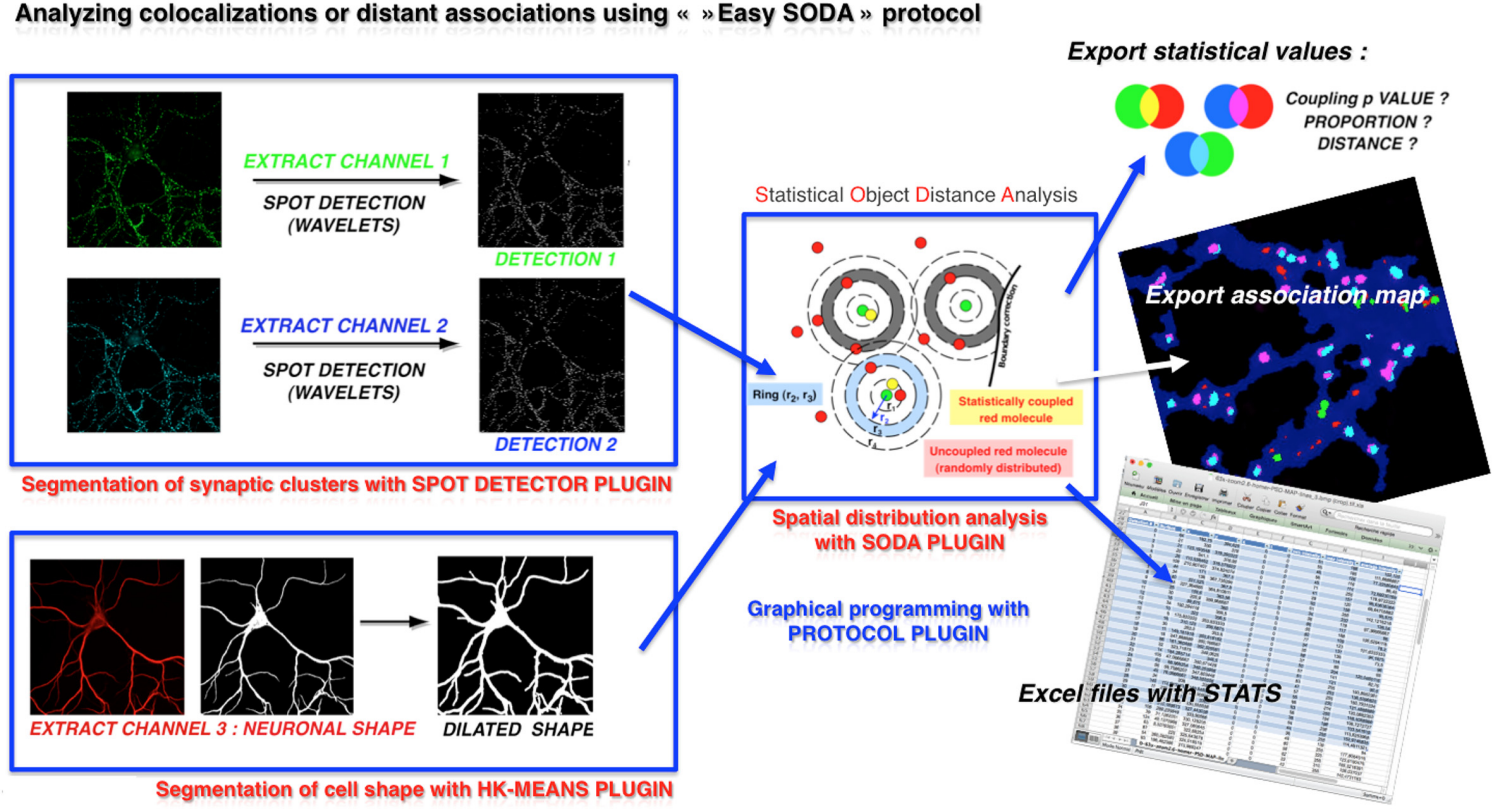
Workflow of the Easy SODA protocol available in Icy Software. Colocalization or distant association can be analyzed using the user-friendly Easy SODA protocol that is freely available in Icy Software. This protocol is a graphical programming automatization routine that allows analysis of synaptic proteins’ distribution within neuronal cell shape. Here, neurons are labeled with two proteins (green and cyan channels) that are distributed in clusters. Clusters are segmented using wavelet segmentation through “spot detector” plugin. Cell shape is extracted using a MAP2 stain, and segmentation is done using the “Hierarchical KMeans” plugin. Cluster distribution within the cellular mask is then analyzed through the “SODA” plugin using Ripley’s analysis. Statistical associations are detected if any, and the proportion of associated clusters with their distance is provided with a p-value indicating the statistical robustness of the association. If many pictures are analyzed in batch mode, all results can be exported to Excel files. A molecular map is exported for each picture with an association color code. For example, if we take a red-green spot analysis: isolated green spots remain green, green spots associated with red are cyan, isolated red spots remain red, and red spots associated with green are pink. Localization of significant associations is thus visible at a glance over the cell mask (here in deep blue).

To analyze the structure of synapses quantitatively, we recently developed, in association with statisticians, free software to detect the association of pre- and postsynaptic proteins. This software, called SODA for standard object distance analysis, makes it possible to identify and measure the spatial distribution of either clusters (conventional microscopy) or single molecules (single molecule localization microscopy) and provides the distance of association when those are statically found associated.^1^^,^^2^ SODA (available here; see Ref. 3) can be used in conventional microscopy (confocal, widefield, and video microscopy) or in super-resolution microscopy, such as SIM, STED, or even SMLM (PALM or STORM). From the mathematical point of view, SODA will analyze the cellular shape and spot density (Fig. 1) to evaluate the expected spatial distribution using Ripley’s function. If clusters are more frequently associated than the expected random distribution, then they are identified as associated spots. As a proof of concept, we analyzed the distribution of three synaptic molecules named synapsin, homer, and PSD-95 using SIM microscopy. Using only 15 pictures, we were able to analyze about 50,000 synapses and identify that the distance between a synapsin and post-synaptic PSD95 cluster was 107±73  nm while the PSD-95-homer was 64±48  nm. Beyond raw distances, this system allows to detect in a quantitative manner any morphological variations that may occur in various mutants, physiological conditions or in certain synaptopathies.

SODA can be used either with sparse labeling (>30−100 objects per image)^4^ or with high-density labeling as in single-molecule localization microscopy^1^^,^^2^ where several thousands of localizations can be retrieved. The only limitation of SODA is the need to get the cell boundary to correctly evaluate the object’s density. In contrast to methods using Voronoï tesselation^5^ that are limited up to now two-dimensional analysis, SODA can be used in 3D, which is an added value to analyze thick 3D volume STORM images. Because SODA does not rely on any overlap methods, it is far less sensitive to high-density false positive colocalization artifacts.^1^^,^^2^

SODA can also be used for all other associations (either direct or distant) in neurosciences^6^^–^^10^ and even outside this field like, for example, in cell biology,^11^^–^^18^ virology,^12^ in bacteria^19^ or plants.^20^ Its use has been highlighted in several reviews.^20^^–^^22^

## 3 Imaging Plasma Membrane in Live or Fixed Cells

### 3.1 Optimizing Live Plasma Membrane Imaging with MemBright Probes

In order to be able to place the molecules within the cell shape, and in collaboration with chemists, we have selected new membrane probes capable of revealing the cell shape and imaging fine structures, such as dendritic spines.^23^

**Fig. 2:**
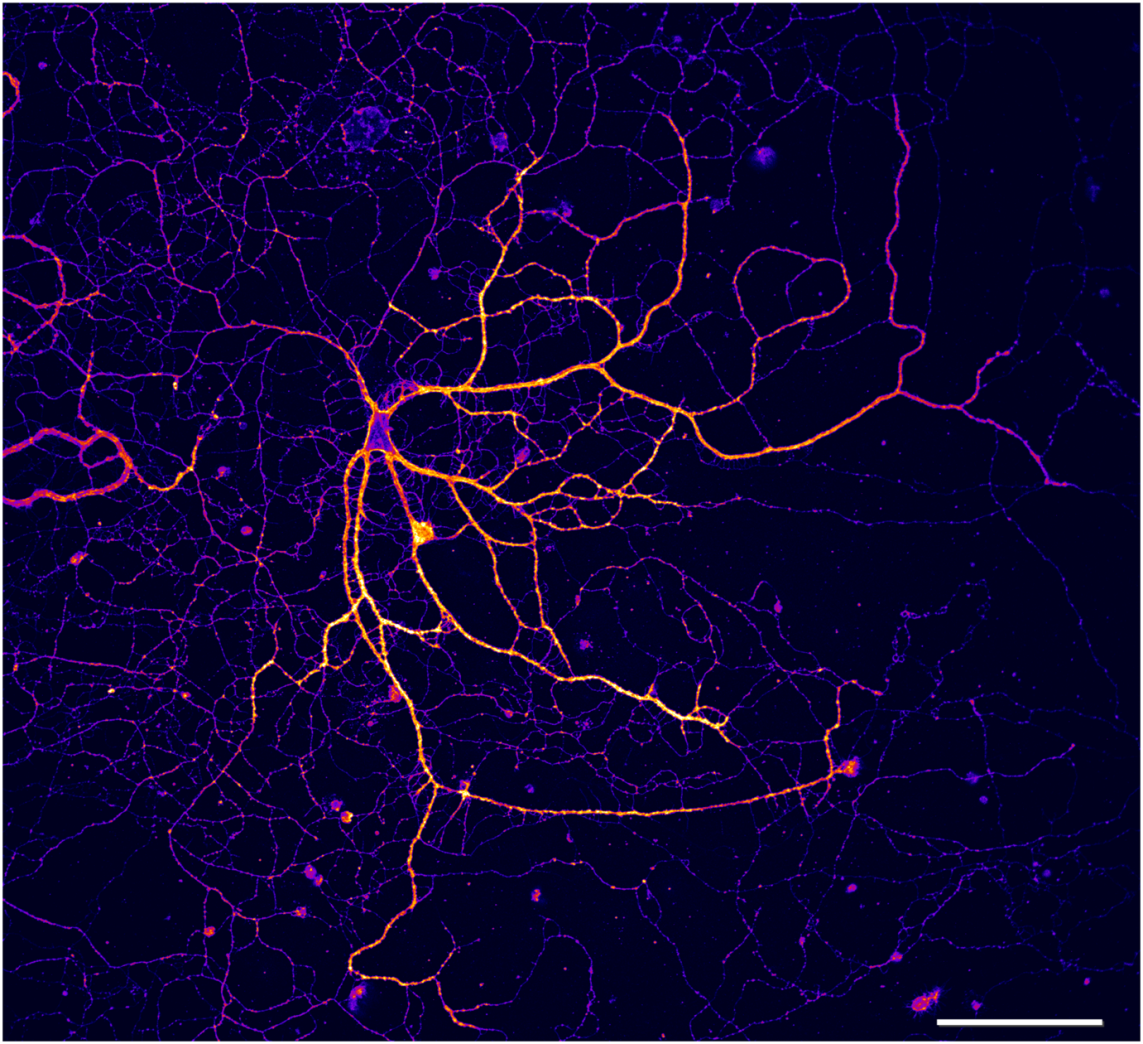
Confocal image (20×) of hippocampal neurons after 2 months in a culture labeled with Cy3.5-MemBright probe reveals dendritic and axonal branch complexity. Scale bar: 100  μm.

These membrane probes, named MemBright, upon insertion in the membrane, emit fluorescence with narrow emission peaks, allowing correlation with other conventional fluorescence for multi-color labeling. A family of seven members is now available and can be used all over the fluorescence spectrum (from 480 and 750 nm). Upon incubation with living cells, these probes insert directly into the membrane through a lipid anchor and thus reveal the cell shapes without the use of any transfection or viral vectors (Fig. 2). This strategy thus makes it possible to reveal all neurons and/or glial cells in a few minutes without any toxicity.

The big advantage of MemBright probes is also its fluorogenic property. Indeed, MemBright probes are non-fluorescent within media and become fluorescent when reaching the plasma membrane. It means that the probe can be let within the cell culture media without having a fluorescence background under the microscope. Letting the probe in the cell chamber during live acquisition allows the perpetual replacement of the probes if bleaching occurs, thus leading to persistent bright labeling of the membrane.

**Fig. 3:**
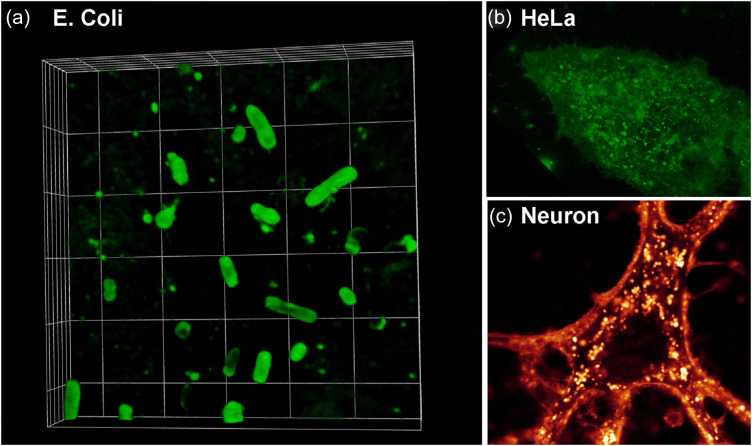
Confocal images (93×) of various cell types labeled with Cy3.5-MemBright probe. (a) 3D rendering of *E.coli* bacteria incubated overnight with MemBright probes. (b) 3D rendering of HeLa cells incubated 30 min with MemBright. (c) Confocal section of hippocampal neurons incubated several hours with MemBright, revealing intracellular vesicles.

MemBright probes are more efficient when incubated on cells in the absence of serum to avoid any titration of the probes by serum fat. In neurons and glial cells, we usually incubate MemBright probes in Krebs Ringer solution at a concentration of 200 nM at 37 deg under the microscope. The absence of serum optimizes the labeling, and the absence of phenol red lowers the fluorescence background. Fluorescence on the plasma membrane appears very fast within a few minutes. To avoid saturation of the plasma membrane with a huge amount of lipids, it is crucial not to use a high concentration of probes. MemBright probes are sufficiently bright to be used at the nanomolar range, whereas other commercial probes have to be used at the micromolar range. Moreover, it should be stressed that illumination of any fluorescent probes may induce the production of reactive oxygen species that can imbalance the intracellular redox state and be deleterious to the cell.^24^ Thus, it is also a good practice to minimize illumination time and frequency, to the minimal amount needed to the right sampling of a biological event. Previously, we could follow neuronal growth over time during 13 h, imaging every 2 min with low laser power (0.2% of an 561 laser line of an Elira PS1), without any detrimental effects.^23^ At last, we have shown that MemBright probes are resistant to permeabilization when fixed properly with a mixture of 4% paraformaldehyde-0.2%glutaraldehyde, and can thus be combined with conventional immunolabeling with primary and secondary antibodies.^23^ MemBright staining can be correlated either to live antibody staining or with immunochemistry on fixed samples. We could show double labeling of the plasma membrane and live L1-CAM endocytosed antibodies. We also showed that intracellular vesicular transporter VGLUT could be revealed by immunochemistry within 3D neuronal cell shape reconstructed using MemBright. This property thus allows identifying surface or internal protein locations using MemBright counterstaining to visualize cell shape. We have selected MemBright probes for their stability at the plasma membrane and their slow endocytosis. However, on long-term incubation, they will be finally endocytosed and can thus be used to label endocytic pathways [Fig. 3(c)].^25^

### 3.2 Imaging Plasma Membrane of Various Cell Types

We originally showed that MemBright probes could be used in various cell types, such as epithelial cells in culture (HeLa cells or KB cells),^23^^,^^26^^–^^28^ and dissociated hippocampal neurons,^23^ hippocampal astrocytes.^23^ We could also use MemBright probes to label live brain (hippocampus, cortex, and cerebellum) or liver slices, allowing the labeling in depth and imaging using confocal or two photons microscopy.^23^ Since our first paper in 2019, MemBright probes have been used by several other labs and cited in more than 60 articles and 39 reviews.^29^^–^^57^ It has been used in B lymphocytes,^58^ in A431 cells,^59^ and reused in neuronal cells to label growth cone and initial segments of hippocampal neurons,^60^ presynaptic terminals,^29^ and post-synaptic compartments.^53^ It has also been used to label apoptotic bodies (AB), microvesicles (MV), and small EV (sEV) isolated from MIN6 pancreatic beta cells exposed to inflammatory, hypoxic, or genotoxic stressors.^61^ Since we were asked several times if MemBright could be used on bacteria, we did recently *E. coli* live labeling with MemBright – CY3.5. As shown in Fig. 3(a), the bacteria 3D shape can be efficiently labeled in live.

### 3.3 Imaging Extracellular Vesicles

MemBright has been widely adopted by the extracellular vesicles community^51^ to track extracellular vesicles both *in vitro* or *in vivo*^61^^–^^81^ in hippocampal^63^ or cortical neurons,^79^ zebrafish,^62^^,^^67^^,^^72^ breast cancer cells or tumours,^66^^,^^71^^,^^80^ myotubes,^82^ and red blood cells.^74^^,^^76^

Particle size distribution and zeta potential analysis of EVs derived from A375 cells using nanoparticle tracking analysis (NTA) showed that EVs labeled before and after labeling by MemBright have almost no change in size and only a slight shift of zeta potential.^75^ Due to its ease of use and brightness, MemBright has thus been widely used to label exosomes. However, it should be stressed that MemBright is not specific to extracellular vesicle labeling. MemBright will be able to label any membrane in contact with the probe. That means that membrane debris trails left behind by migrating cells will be labeled, whatever the nature of the membrane (EVs or not). Any membranous organelles (tubules, endosomes, lysosomes, synaptosomes, etc.) that would have been retrieved by ultracentrifugation can be labeled when incubated with MemBright. Some controls are thus needed before labeling to ensure that the fraction is homogeneous and not contaminated by different organelles.

Hyenne et al.^62^ show that MemBright can be used in pulse-chase experiments and that some CD63-GFP EVs can be labeled with MemBright, while others are not.^62^ Sung et al. concluded that MemBright can label exosomes as well as plasma membrane-derived EVs, but that MemBright does not label all exosomes.^47^ Using a pulse-chase experiment, it is expected that not all endosomal compartments will be labeled. Indeed, only those deriving from the plasma membrane exposed to the MemBright at a time t will be visible. It is likely that a proportion of EVs that were generated before or after incubation with MemBright and that are then stored in the cell will not be labeled by the MemBright wave. It is therefore essential to properly calibrate the labeling time incubation and the chase time to observe the desired events.

## 4 Imaging Plasma Membrane with Single Molecule Localization Microscopy

**Fig. 4:**
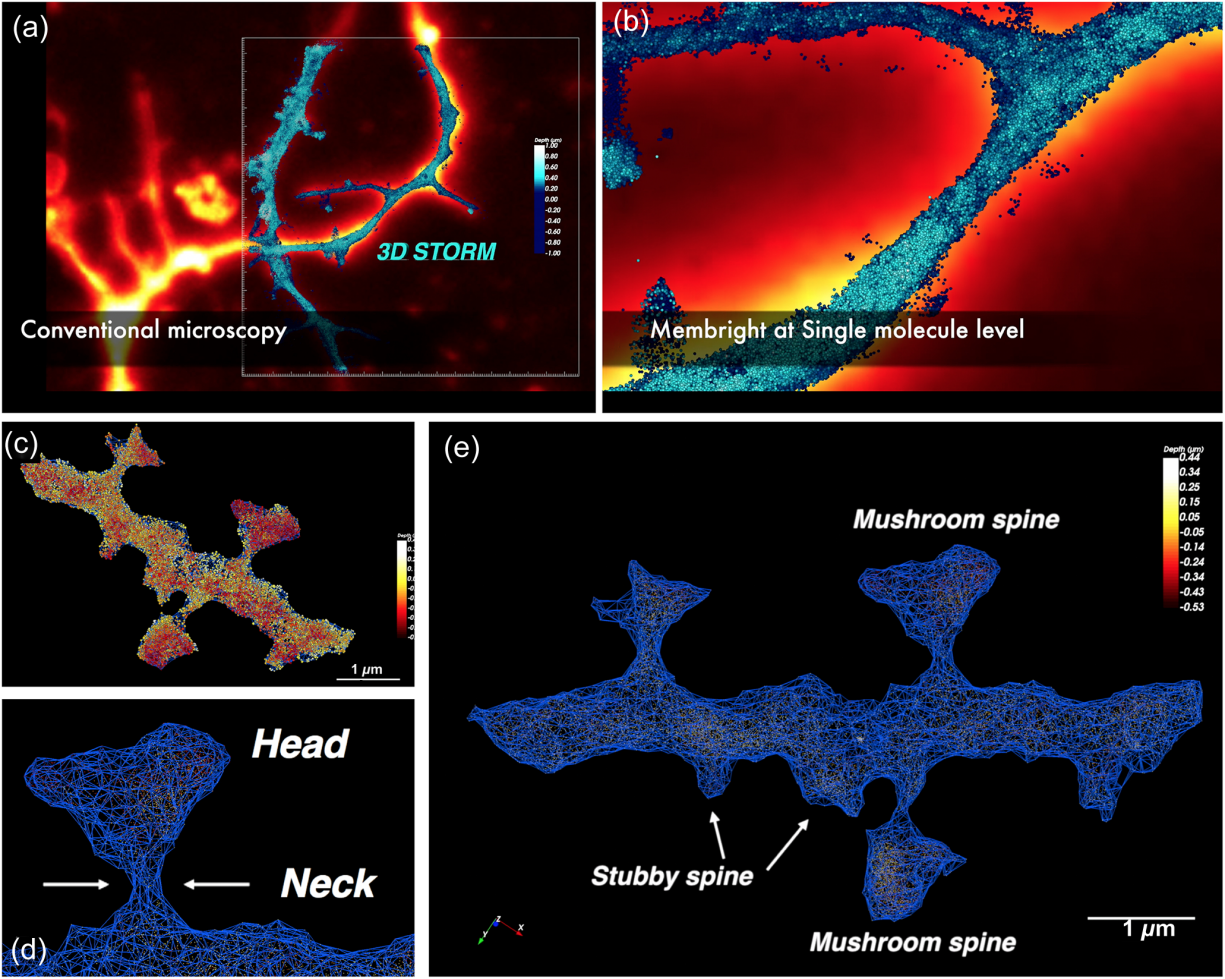
STORM imaging of hippocampal neurons labeled with Cy3.5-MemBright probe and imaged in conventional widefield microscopy [fire LUT in (a)] and in STORM microscopy (blue spheres in rectangle). (b) Magnification of the plasma membrane 3D STORM image shows the single molecule organization of MemBright all over the plasma membrane. Light blue localizations are closer and deep blue are deeper. (c)–(e) Example of stubby and mushroom dendritic spines in 3D STORM. All the localizations found in (c) (published previously in a different form in Ref. 3) can be used to reconstruct the 3D shape of the spine in a wireframe. This 3D shape can then be used for volumetric estimation or fine measurements of the spine neck.

MemBright probes can be used in conventional and super-resolution microscopy (Fig. 4 and Video 1) and thus make it possible to observe the molecular distribution of synaptic proteins in correlation with the structural morphology at the nanoscale using STORM. Altogether with the SODA plugin, MemBright probes thus provide the perfect tools to access a nanometric molecular map of various proteins within the 3D cellular context. These high-resolution techniques were set up on fixed samples, but it is expected that the need for super-resolution imaging with small molecular probes to allow imaging of living samples will be growing and will stimulate the development of new molecular probes. Indeed, chemical development will be required in the next years, since molecular probes are still up to now far beyond the recent development in instrumentation that can reach a resolution of 3 to 4 nm.^83^ One interesting track will be probably the development of self-blinking dyes^84^^–^^86^ or the development of new convertible fluorophores^28^ for live SMLM. We are now working on a photoswitchable version of the MemBright that would be able to be photoswitched without any reducing buffer, to be used in live single molecule localization microscopy.^87^ Moreover since MemBright has been used a lot in the extracellular vesicle community, it indicates that membrane dyes are of great interest for people working on vesicular trafficking. One other challenge, in the next years, would be to develop new MemBright probes devoted to fast internalization, to be able to decipher different vesicular pathways with various colors.

## 5 Appendix: Supplementary Information

Video 1 Conventional and STORM imaging of hippocampal neurons labeled with Cy3.5-MemBright to label dendritic spines in 3D at the nanoscale level (MP4, 60.9 MB [URL: https://doi.org/10.1117/1.NPh.11.1.014414.s1]).

## Supplementary Material



## Data Availability

Tutorial and download concerning SODA plugin and user-friendly “Easy SODA protocol,” can be found on Icy Software website here: https://icy.bioimageanalysis.org/protocol/easy-soda-2-colors-1-image/ Sample image is also available here on Zenodo: https://zenodo.org/record/4323312-.Y_3tqx1CdHQ A French Tutorial on Ripley’s function and Icy SODA plugin can be found on YouTube: https://www.youtube.com/watch?v=7yVp73s-4TA

## References

[1] Lagache T. et al., “Mapping molecular assemblies with fluorescence microscopy and object-based spatial statistics,” Nat. Commun. 9(1), 698 (2018). 10.1038/s41467-018-03053-x 29449608 PMC5814551

[2] Lagache T. et al., “Statistical analysis of molecule colocalization in bioimaging,” Cytometry A 87(6), 568–579 (2015). 10.1002/cyto.a.22629 25605428

[3] https://icy.bioimageanalysis.org/.

[4] Lagache T. Meas-Yedid V. Olivo-Marin J. C., “A statistical analysis of spatial colocalization using Ripley’s K function,” in IEEE 10th Int. Symp. Biomed. Imaging, pp. 896–901 (2013). 10.1109/ISBI.2013.6556620

[5] Levet F. et al., “SR-Tesseler: a method to segment and quantify localization-based super-resolution microscopy data,” Nat. Methods 12(11), 1065–1071 (2015). 10.1038/nmeth.3579 26344046

[6] Badawi Y. Nishimune H., “Super-resolution microscopy for analyzing neuromuscular junctions and synapses,” Neurosci. Lett. 715, 134644 (2020). 10.1016/j.neulet.2019.134644 31765730 PMC6937598

[r7] Bodzeta A. Berger F. MacGillavry H. D., “Subsynaptic mobility of presynaptic mGluR types is differentially regulated by intra- and extracellular interactions,” Mol. Biol. Cell 33(8), ar66 (2022). 10.1091/mbc.E21-10-0484 35511883 PMC9635276

[r8] Gagliano G. et al., “Light sheet illumination for 3D single-molecule super-resolution imaging of neuronal synapses,” Front. Synaptic Neurosci. 13, 761530 (2021). 10.3389/fnsyn.2021.761530 34899261 PMC8651567

[r9] Gallo A. et al., “Role of the Sec22b-E-Syt complex in neurite growth and ramification,” J. Cell Sci. 133(18), jcs247148 (2020). 10.1242/jcs.247148 32843578

[10] Lenoir S. et al., “Pridopidine rescues BDNF/TrkB trafficking dynamics and synapse homeostasis in a Huntington disease brain-on-a-chip model,” Neurobiol. Dis. 173, 105857 (2022). 10.1016/j.nbd.2022.105857 36075537

[11] Burrinha T. et al., “Upregulation of APP endocytosis by neuronal aging drives amyloid-dependent synapse loss,” J. Cell Sci. 134(9), jcs255752 (2021). 10.1242/jcs.255752 33910234

[r12] Cosentino G. et al., “Respiratory syncytial virus ribonucleoproteins hijack microtubule Rab11 dependent transport for intracellular trafficking,” PLoS Pathogens 18(7), e1010619 (2022). 10.1371/journal.ppat.1010619 35797399 PMC9262236

[r13] Ferrari M. L. et al., “*Shigella* promotes major alteration of gut epithelial physiology and tissue invasion by shutting off host intracellular transport,” Proc. Natl. Acad. Sci. U. S. A. 116(27), 13582–13591 (2019). 10.1073/pnas.1902922116 31209035 PMC6613108

[r14] Cristofari P. et al., “Nanoscopic distribution of VAChT and VGLUT3 in striatal cholinergic varicosities suggests colocalization and segregation of the two transporters in synaptic vesicles,” Front. Mol. Neurosci. 15, 991732 (2022). 10.3389/fnmol.2022.991732 36176961 PMC9513193

[r15] Grassart A. et al., “Bioengineered human organ-on-chip reveals intestinal microenvironment and mechanical forces impacting *Shigella* infection,” Cell Host Microbe 26(3), 435–444.e4 (2019). 10.1016/j.chom.2019.08.007 31492657

[r16] Gu B. et al., “Opposing effects of cohesin and transcription on CTCF organization revealed by super-resolution imaging,” Mol. Cell 80(4), 699–711.e7 (2020). 10.1016/j.molcel.2020.10.001 33091336 PMC7725164

[r17] Kamiyama R. et al., “Cell-type-specific, multicolor labeling of endogenous proteins with split fluorescent protein tags in *Drosophila*,” Proc. Natl. Acad. Sci. U. S. A. 118(23), e2024690118 (2021). 10.1073/pnas.2024690118 34074768 PMC8201798

[18] Walpole G. F. W. et al., “Inactivation of Rho GTPases by *Burkholderia cenocepacia* induces a WASH-mediated actin polymerization that delays phagosome maturation,” Cell Rep. 31(9), 107721 (2020). 10.1016/j.celrep.2020.107721 32492429 PMC7315377

[19] Altinoglu I. Merrifield C. J. Yamaichi Y., “Single molecule super-resolution imaging of bacterial cell pole proteins with high-throughput quantitative analysis pipeline,” Sci. Rep. 9, 6680 (2019). 10.1038/s41598-019-43051-7 31040310 PMC6491441

[20] Colin L. et al., “Imaging the living plant cell: from probes to quantification,” Plant Cell 34(1), 247–272 (2022). 10.1093/plcell/koab237 34586412 PMC8774089

[r21] Khan A. O. Pike J. A., “Super-resolution imaging and quantification of megakaryocytes and platelets,” Platelets 31(5), 559–569 (2020). 10.1080/09537104.2020.1732321 32079444

[22] Mancebo A. et al., “Efficient cross-correlation filtering of one- and two-color single molecule localization microscopy data,” Front. Bioinf. 1, 739769 (2021). 10.3389/fbinf.2021.739769 PMC9581065 36303727

[23] Collot M. et al., “MemBright: a family of fluorescent membrane probes for advanced cellular imaging and neuroscience,” Cell Chem. Biol. 26(4), 600–614.e7 (2019). *corresponding authors. 10.1016/j.chembiol.2019.01.009 30745238

[24] Marchetti M. et al., “Genetically encoded biosensors for the fluorescence detection of O2 and reactive O2 species,” Sensors 23(20), 8517 (2023). 10.3390/s23208517 37896609 PMC10611200

[25] Collot M. et al., “Probing polarity and heterogeneity of lipid droplets in live cells using a push-pull fluorophore,” Anal. Chem. 91(3), 1928–1935 (2019). 10.1021/acs.analchem.8b04218 30592219

[26] Michelis S. et al., “Imaging and measuring vesicular acidification with a plasma membrane-targeted ratiometric pH probe,” Anal. Chem. 94(15), 5996–6003 (2022). 10.1021/acs.analchem.2c00574 35377610

[r27] Collot M. et al., “Ultrabright and fluorogenic probes for multicolor imaging and tracking of lipid droplets in cells and tissues,” J. Am. Chem. Soc. 140(16), 5401–5411 (2018). 10.1021/jacs.7b12817 29446627

[28] Saladin L. et al., “Dual-color photoconvertible fluorescent probes based on directed photooxidation induced conversion for bioimaging,” Angew. Chem. Int. Ed. Engl. 62(4), e202215085 (2023). 10.1002/anie.202215085 36420823 PMC10107923

[29] Harper C. B. Smillie K. J., “Current molecular approaches to investigate pre-synaptic dysfunction,” J. Neurochem. 157(2), 107–129 (2021). 10.1111/jnc.15316 33544872

[r30] Hu K. K. et al., “Chemical analysis of single cells and organelles,” Anal. Chem. 93(1), 41–71 (2021). 10.1021/acs.analchem.0c04361 33284577 PMC7807422

[r31] Jia H. R. et al., “Cell surface-localized imaging and sensing,” Chem. Soc. Rev. 50(10), 6240–6277 (2021). 10.1039/D1CS00067E 34027939

[r32] Kikuchi K. et al., “Photochemical mechanisms of fluorophores employed in single-molecule localization microscopy,” Angew. Chem. Int. Edit. 62(1), 25 (2023). 10.1002/anie.202204745 PMC10100239 36177530

[r33] Kim H. J. Jo S. H., “Nighttime administration of antihypertensive medication: a review of chronotherapy in hypertension,” Kor. J. Intern. Med. 10, 10 (2023). 10.3904/kjim.2023.304 PMC10918378 37967524

[r34] Klymchenko A. S., “Fluorescent probes for lipid membranes: from the cell surface to organelles,” Accounts Chem. Res. 56(1), 1–12 (2023). 10.1021/acs.accounts.2c00586 36533992

[r35] Kooijmans S. A. A. de Jong O. G. Schiffelers R. M., “Exploring interactions between extracellular vesicles and cells for innovative drug delivery system design,” Adv. Drug Deliv. Rev. 173, 252–278 (2021). 10.1016/j.addr.2021.03.017 33798644

[r36] Kundu R. Chandra A. Datta A., “Fluorescent chemical tools for tracking anionic phospholipids,” Isr. J. Chem. 61(3–4), 199–216 (2021). 10.1002/ijch.202100003

[r37] Liu C. L. et al., “Advances in the development of fluorescence probes for cell plasma membrane imaging,” Trends Anal. Chem. 133, 116092 (2020). 10.1016/j.trac.2020.116092

[r38] Magliaro C. et al., “Gotta Trace ’em All: a mini-review on tools and procedures for segmenting single neurons toward deciphering the structural connectome,” Front. Bioeng. Biotechnol. 7(8), 202 (2019). 10.3389/fbioe.2019.00202 31555642 PMC6727034

[r39] Mansuri S. Mukherjee T. Kanvah S., “Fluorescent sterol probes for intracellular transport, imaging, and therapeutics,” Curr. Opin. Chem. Biol. 71(8), 102222 (2022). 10.1016/j.cbpa.2022.102222 36219959

[r40] Méndez-Ardoy A. Reina J. J. Montenegro J., “Synthesis and supramolecular functional assemblies of ratiometric pH probes,” Chem. Eur. J. 26(34), 7516–7536 (2020). 10.1002/chem.201904834 31945215

[r41] Mukherjee S. et al., “Imaging viral infection by fluorescence microscopy: focus on HIV-1 early stage,” Viruses 13(2), 213 (2021). 10.3390/v13020213 33573241 PMC7911428

[r42] Pivovarenko V. G. Klymchenko A. S., “Fluorescent probes based on charge and proton transfer for probing biomolecular environment,” Chem. Rec. 30, e202300321 (2023). 10.1002/tcr.202300321 38158338

[r43] Salomon C. et al., “Extracellular vesicles and their emerging roles as cellular messengers in endocrinology: an endocrine society scientific statement,” Endocr. Rev. 43(3), 441–468 (2022). 10.1210/endrev/bnac009 35552682 PMC10686249

[r44] Samanta S. et al., “Xanthene, cyanine, oxazine and BODIPY: the four pillars of the fluorophore empire for super-resolution bioimaging,” Chem. Soc. Rev. 52(20), 7197–7261 (2023). 10.1039/D2CS00905F 37743716

[r45] Schneider F. Colin-York H. Fritzsche M., “Quantitative bio-imaging tools to dissect the interplay of membrane and cytoskeletal actin dynamics in immune cells,” Front. Immunol. 11(13), 612542 (2021). 10.3389/fimmu.2020.612542 33505401 PMC7829180

[r46] Sun Y. et al., “Single-molecule detection-based super-resolution imaging in single-cell analysis: Inspiring progress and future prospects,” Trends Anal. Chem. 167(18), 117255 (2023). 10.1016/j.trac.2023.117255

[r47] Sung B. H. Parent C. A. Weaver A. M., “Extracellular vesicles: critical players during cell migration,” Dev. Cell 56(13), 1861–1874 (2021). 10.1016/j.devcel.2021.03.020 33811804 PMC8282723

[r48] Sych T. et al., “How does liquid-liquid phase separation in model membranes reflect cell membrane heterogeneity?” Membranes 11(5), 323 (2021). 10.3390/membranes11050323 33925240 PMC8146956

[r49] Tosheva K. L. et al., “Between life and death: strategies to reduce phototoxicity in super-resolution microscopy,” J. Phys. D-Appl. Phys. 53(16), 163001 (2020). 10.1088/1361-6463/ab6b95 33994582 PMC8114953

[r50] Ural E. E. et al., “Visualizing extracellular vesicles and their function in 3D tumor microenvironment models,” Int. J. Mol. Sci. 22(9), 4784 (2021). 10.3390/ijms22094784 33946403 PMC8125158

[r51] Verweij F. J. et al., “The power of imaging to understand extracellular vesicle biology *in vivo*,” Nat. Methods 18(9), 1013–1026 (2021). 10.1038/s41592-021-01206-3 34446922 PMC8796660

[r52] Welsh J. A. et al., “A compendium of single extracellular vesicle flow cytometry,” J. Extracell. Vesicles 12(2), 70 (2023). 10.1002/jev2.12299 PMC9911638 36759917

[r53] Werner C. Sauer M. Geis C., “Super-resolving microscopy in neuroscience,” Chem. Rev. 121(19), 11971–12015 (2021). 10.1021/acs.chemrev.0c01174 33752325

[r54] Xing Y. L. et al., “Analysis of extracellular vesicles as emerging theranostic nanoplatforms,” Coord. Chem. Rev. 424, 213506 (2020). 10.1016/j.ccr.2020.213506

[r55] Xu S. et al., “Molecular engineering of near-infrared fluorescent probes for cell membrane imaging,” Molecules 28(4), 1906 (2023). 10.3390/molecules28041906 36838896 PMC9960866

[r56] Yang L. et al., “Small-molecule fluorescent probes for plasma membrane staining: design, mechanisms and biological applications,” Coord. Chem. Rev. 474, 214862 (2023). 10.1016/j.ccr.2022.214862

[57] Zhou C. Chia G. W. N. Yong K. T., “Membrane-intercalating conjugated oligoelectrolytes,” Chem. Soc. Rev. 51(24), 9917–9932 (2022). 10.1039/D2CS00014H 36448452

[58] Kwon H. Y. et al., “Lipid-oriented live-cell distinction of B and T lymphocytes,” J. Am. Chem. Soc. 143(15), 5836–5844 (2021). 10.1021/jacs.1c00944 33834782

[59] Charpentier C. et al., “Ultrabright terbium nanoparticles for FRET biosensing and in situ imaging of epidermal growth factor receptors,” Chem. Eur. J. 26(64), 14602–14611 (2020). 10.1002/chem.202002007 32501573

[60] Honda A. et al., “Very-long-chain fatty acids are crucial to neuronal polarity by providing sphingolipids to lipid rafts,” Cell Rep. 42(10), 113195 (2023). 10.1016/j.celrep.2023.113195 37816355

[61] Giri K. R. et al., “Molecular and functional diversity of distinct subpopulations of the stressed insulin-secreting cell’s vesiculome,” Front. Immunol. 11, 1814 (2020). 10.3389/fimmu.2020.01814 33101266 PMC7556286

[r62] Hyenne V. et al., “Studying the fate of tumor extracellular vesicles at high spatiotemporal resolution using the zebrafish embryo,” Dev. Cell 48(4), 554–572.e7 (2019). 10.1016/j.devcel.2019.01.014 30745140

[r63] Antoniou A. et al., “Neuronal extracellular vesicles and associated microRNAs induce circuit connectivity downstream BDNF,” Cell Rep. 42(2), 112063 (2023). 10.1016/j.celrep.2023.112063 36753414

[r64] Boyer M. J. et al., “Endothelial cell-derived extracellular vesicles alter vascular smooth muscle cell phenotype through high-mobility group box proteins,” J. Extracell. Vesicles 9(1), 19 (2020). 10.1080/20013078.2020.1781427 PMC7480479 32944170

[r65] Dehghani M. et al., “Systematic evaluation of PKH labelling on extracellular vesicle size by nanoparticle tracking analysis,” Sci. Rep. 10(1), 10 (2020). 10.1038/s41598-020-66434-7 32533028 PMC7293335

[r66] Gargiulo E. et al., “Extracellular vesicle secretion by leukemia cells *in vivo* promotes CLL progression by hampering antitumor t-cell responses,” Blood Cancer Discov. 4(1), 54–77 (2023). 10.1158/2643-3230.BCD-22-0029 36108149 PMC9816815

[r67] Ghoroghi S. et al., “Ral GTPases promote breast cancer metastasis by controlling biogenesis and organ targeting of exosomes,” Elife 10, e61539 (2021). 10.7554/eLife.61539 33404012 PMC7822591

[r68] He Y. et al., “Fluorescence labeling of extracellular vesicles for diverse bio-applications *in vitro* and *in vivo*,” Chem. Commun. 59(44), 6609–6626 (2023). 10.1039/D3CC00998J 37161668

[r69] Jung S. R. et al., “Error-correction method for high-throughput sizing of nanoscale vesicles with single-molecule localization microscopy,” J. Phys. Chem. B 127(12), 2701–2707 (2023). 10.1021/acs.jpcb.2c09053 36944080 PMC10224584

[r70] Lees R. et al., “Single extracellular vesicle transmembrane protein characterization by nano-flow cytometry,” J. Vis. Exp. (185), 15 (2022). 10.3791/64020 35969098

[r71] Loconte L. et al., “Detection of the interactions of tumour derived extracellular vesicles with immune cells is dependent on EV-labelling methods,” J. Extracell. Vesicles 12(12), 15 (2023). 10.1002/jev2.12384 PMC10687762 38031976

[r72] Mary B. et al., “Blood flow diverts extracellular vesicles from endothelial degradative compartments to promote angiogenesis,” EMBO Rep. 24, e57042 (2023). 10.15252/embr.202357042 37971863 PMC10702841

[r73] Melling G. E. et al., “Confocal microscopy analysis reveals that only a small proportion of extracellular vesicles are successfully labelled with commonly utilised staining methods,” Sci. Rep. 12(1), 13 (2022). 10.1038/s41598-021-04225-4 34997141 PMC8741769

[r74] Musicò A. et al., “Surface functionalization of extracellular vesicle nanoparticles with antibodies: a first study on the protein corona “variable”,” Nanoscale Adv. 5(18), 4703–4717 (2023). 10.1039/D3NA00280B 37705771 PMC10496878

[r75] Ren Y. A. et al., “Rapid enrichment and sensitive detection of extracellular vesicles through measuring the phospholipids and transmembrane protein in a microfluidic chip,” Biosens. Bioelectron. 199, 113870 (2022). 10.1016/j.bios.2021.113870 34915212

[r76] Rodriguez B. V. et al., “An *ex vivo* model of interactions between extracellular vesicles and peripheral mononuclear blood cells in whole blood,” J. Extracell. Vesicles 12(12), 12368 (2023). 10.1002/jev2.12368 38047476 PMC10694845

[r77] Sato Y. Ohira K. Nishizawa S., “Self-assembly and disassembly of membrane curvature-sensing peptide-based deep-red fluorescent probe for highly sensitive sensing of exosomes,” ACS Sens. 5, 522–526 (2023). 10.1021/acssensors.2c02498 36695520

[r78] Shimomura T. et al., “New lipophilic fluorescent dyes for labeling extracellular vesicles: characterization and monitoring of cellular uptake,” Bioconjugate Chem. 32(4), 680–684 (2021). 10.1021/acs.bioconjchem.1c00068 33719402

[r79] Solana-Balaguer J. et al., “Neuron-derived extracellular vesicles contain synaptic proteins, promote spine formation, activate TrkB-mediated signalling and preserve neuronal complexity,” J. Extracell. Vesicles 12(9), e12355 (2023). 10.1002/jev2.12355 37743539 PMC10518375

[r80] Tkach M. et al., “Extracellular vesicles from triple negative breast cancer promote pro-inflammatory macrophages associated with better clinical outcome,” Proc. Natl. Acad. Sci. U. S. A. 119(17), e2107394119 (2022). 10.1073/pnas.2107394119 35439048 PMC9169908

[81] Zhu J. Y. Bazan G. C., “Molecular orientation and optimization of membrane dyes based on conjugated oligoelectrolytes,” Cell Rep. Phys. Sci. 4(6), 101429 (2023). 10.1016/j.xcrp.2023.101429

[82] Lemerle E. et al., “Caveolae and Bin1 form ring-shaped platforms for T-tubule initiation,” Elife 12, e84139 (2023). 10.7554/eLife.84139 37083699 PMC10281672

[83] Schmidt R. et al., “MINFLUX nanometer-scale 3D imaging and microsecond-range tracking on a common fluorescence microscope,” Nat. Commun. 12(1), 1478 (2021). 10.1038/s41467-021-21652-z 33674570 PMC7935904

[84] Remmel M. et al., “Accelerated MINFLUX nanoscopy, through spontaneously fast-blinking fluorophores,” Small 19(12), e2206026 (2023). 10.1002/smll.202206026 36642798

[r85] Remmel M. et al., “Spontaneously blinking fluorophores for accelerated MINFLUX nanoscopy,” bioRxiv 2022.2008.2029.505670 (2022). 10.1002/smll.202206026 36642798

[86] Grussmayer K. et al., “Self-blinking dyes unlock high-order and multiplane super-resolution optical fluctuation imaging,” ACS Nano 14(7), 9156–9165 (2020). 10.1021/acsnano.0c04602 32567836

[87] Saladin L. et al., “Targeted photoconvertible BODIPYs based on directed photooxidation induced conversion for applications in photoconversion and live super resolution imaging,” bioRxiv 2023.2007.2028.550940 (2023). 10.1021/jacs.4c05231 38861358

